# MRI of acute neck infections: evidence summary and pictorial review

**DOI:** 10.1186/s13244-022-01347-9

**Published:** 2023-01-08

**Authors:** Jussi Hirvonen, Jaakko Heikkinen, Mikko Nyman, Tatu Happonen, Jarno Velhonoja, Heikki Irjala, Tero Soukka, Kimmo Mattila, Janne Nurminen

**Affiliations:** 1 Department of Radiology, University of Turku and Turku University Hospital, Turku, Finland; 2 Medical Imaging Center, Department of Radiology, Tampere University and Tampere University Hospital, Tampere, Finland; 3 Department of Otorhinolaryngology-Head and Neck Surgery, University of Turku and Turku University Hospital, Turku, Finland; 4 Department of Oral and Maxillofacial Surgery, University of Turku, Turku, Finland

**Keywords:** Magnetic resonance imaging, Neck, Emergency medicine, Infection

## Abstract

Infection of the deep neck spaces is a life-threatening acute illness that requires prompt diagnosis and treatment. Magnetic resonance imaging (MRI) offers unsurpassed soft tissue discrimination and is therefore well suited for imaging neck infections. Recently, the feasibility, diagnostic accuracy, and clinical significance of this method have been documented in patients with acute neck infections. This review article summarizes the scientific evidence, provides a practical guide to image acquisition and interpretation, reviews the most common imaging findings, and discusses some difficult diagnoses and pitfalls in acute neck infections, to help both radiologists and clinicians in managing these critically ill patients.

## Key points

- MRI of acute neck infections is feasible in most patients.
- Most common types are pharyngotonsillar and odontogenic infections.
- MRI has high diagnostic accuracy for abscesses in adults and children.
- MRI shows reproducible edema patterns that have clinical and prognostic significance.

## Background

Deep neck infections are associated with a high complication rate and significant mortality [1]. Aggressive treatment with intravenous antibiotics and surgical drainage of abscesses is usually required. The exact origin, location, and extent of infection may be difficult to define accurately on clinical examination. Therefore, medical imaging has a significant role in diagnosing and treating these patients. The main roles of imaging are to confirm the diagnosis, provide relevant differential diagnoses, demonstrate the presence, location, and extent of surgically drainable abscesses, and reveal the origin of the infection.

Emergency neck imaging has traditionally been performed using contrast-enhanced computed tomography (CT). However, limited soft tissue contrast, uncertainty in separating purulent from non-purulent fluid collections, and artifacts from bone and dental implants may compromise accurate delineation of neck disease. More recently, magnetic resonance imaging (MRI) has proven to be a feasible, accurate, and reliable first-line imaging method in deep neck infections [2]. MRI has excellent soft tissue discrimination and does not involve ionizing radiation.

The purpose of this pictorial review is to provide a comprehensive array of MRI studies of the most commonly encountered deep neck space infections. We will review patient selection, MRI sequences and protocoling, pertinent normal radiological anatomy, typical edema patterns, and imaging patterns of various types of abscesses with important caveats. After reading this review, radiologists should be able to confidently detect and characterize various MRI findings in deep neck infections and describe to the referring clinician the pertinent findings. Because the focus of this review is on the soft tissues of the deep neck spaces, we will not review imaging of infections originating from the orbit, paranasal sinuses, temporal bone, or spine, nor will we review intracranial complications of head and neck infections.

## A brief comparison of imaging methods

**Fig. 1:**
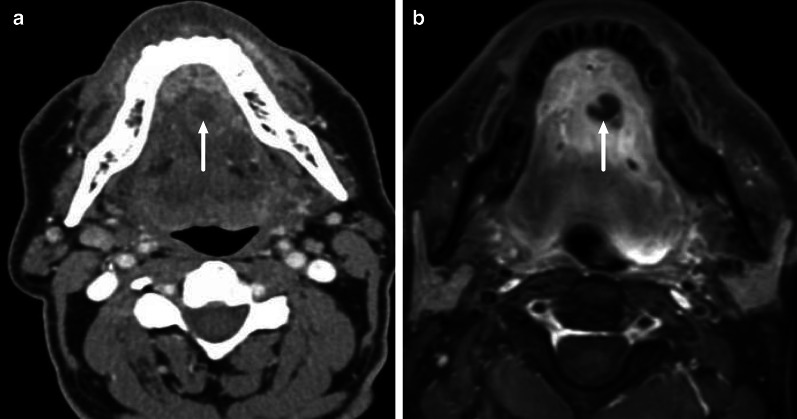
The CT diagnosis of an abscess may be difficult. Images show an abscess in the floor of the mouth on axial contrast-enhanced CT (**a**), and on axial T1-weighted fat-suppressed MRI after gadolinium (**b**) from the same patient imaged within 24 h with both modalities. Subtle central hypodensity and faint rim enhancement can be seen on CT (**a**); this was, however, misinterpreted as cellulitis. A fluid collection is clearly defined within abnormal tissue enhancement on MRI (**b**), and diffusion-weighed imaging (DWI, not shown here) suggested purulence, which was confirmed by surgery

In clinical practice, contrast-enhanced CT is considered the standard imaging modality in the emergency department because of its good availability, fast scanning times, and reasonably low cost [3]. In many patients, CT shows the source and extent of the infection, and potential complications, and thereby provides critical information for surgical planning. There are several diagnostic criteria for the diagnosis of an abscess, including low-density core, rim enhancement, bulging, or scalloping [4]. A limitation of CT is the substantially variable diagnostic accuracy [5–7], likely due to limited soft tissue discrimination. In the early phases of abscess formation, specific diagnostic signs may be subtle and difficult to detect (Fig. 1).

MRI is a promising alternative modality to imaging acute neck infections [2]. The most important advantages of MRI are superior soft tissue discrimination and lack of ionizing radiation. Soft tissue discrimination allows the accurate delineation of normal tissues (such as muscles, fat, glandular tissue, lymphoid tissue, and mucosa), highly sensitive detection of soft tissue and bone marrow edema, and delineation and characterization of abscesses and other collections. The lack of ionizing radiation is particularly beneficial in children and young adults. In our opinion, for experienced readers, MRI studies are also usually faster to read than CT because of higher confidence in relevant findings. Disadvantages of MRI include low availability, longer scanning times, initial challenges for radiologists and clinicians in learning to read the images, and technical challenges in extending the imaging field of view caudally into the mediastinum.

## Emergency MRI in clinical practice

### Patient selection and feasibility

According to a study based on a large cohort, emergency MRI is feasible in almost all patients [2], with an overall success rate of 95% (including both planned and initiated MRI scans). Only a few MRI scans among acutely ill patients (about 1%) were found non-diagnostic as patient restlessness had deteriorated image quality. Some patient-related contraindications may favor the use of CT instead of MRI, such as ferromagnetic foreign objects, restlessness, claustrophobia, high fever, and difficult dyspnea. In addition to adults, the high feasibility and equal diagnostic accuracy of emergency MRI have been demonstrated in children [8].

### Sequences and protocols

At our institution, we have used the Philips 3 Tesla system (Ingenia). The routine protocol includes seven sequences and is completed in about 30 min (Table 1).

**Table 1:** MRI protocol for acute neck infections

Sequence	Gd contrast	Orientation	Purpose
T2 Dixon	No	Axial, coronal	Normal anatomy (in-phase), tissue edema (water image)
T1 SE	No	Axial	Normal anatomy, especially fat pads
DWI	No	Axial	Detection of hypercellularity in solid tissue (normal lymph nodes, tumors), and purulence (abscess formation) in fluid collections
T1 Dixon	Yes	Axial, coronal, sagittal	Demonstration of abnormal tissue enhancement (cellulitis, phlegmon), and indirect demonstration of non-enhancement in fluid collections and necrosis

**Fig. 2:**
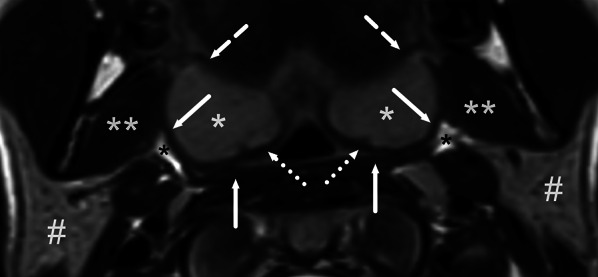
Normal MRI anatomy of the oropharynx. Axial T2-weighted image shows the palatine tonsils (white asterisk) bounded laterally by the superior constrictor muscle (arrow), anteriorly by the palatoglossus muscle (dashed arrow), and posteriorly by the palatopharyngeus muscle (dotted arrow). Posterolateral to the superior constrictor muscle and the buccopharyngeal fascia is the parapharyngeal space indicated by fat signal (black asterisk). The medial pterygoid muscle (double white asterisk) and the parotid gland (hash) can be seen in the masticator and parotid spaces, respectively

**Fig. 3:**
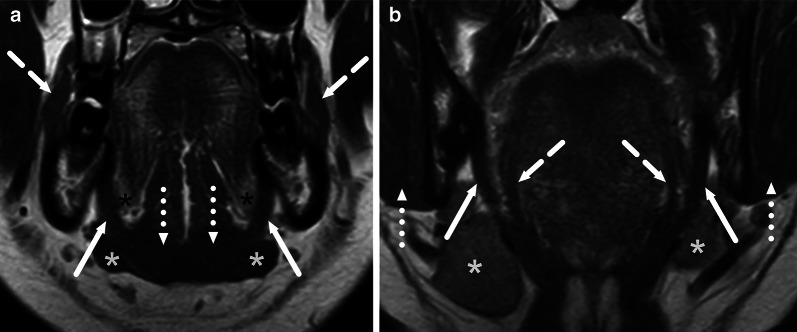
Normal MRI anatomy of the oral cavity. **a** A coronal T2-weighted image at the anterior part shows the mylohyoid muscle (arrow), separating the sublingual space superiorly including the sublingual gland (black asterisk) and the submandibular space inferiorly. Medially, and superior to the mylohyoid muscle, are the geniohyoid muscles (dotted arrow) and genioglossus muscles (superior to the geniohyoid muscles). Inferiorly, images show the anterior belly of the digastric muscle (white asterisk), and laterally, the buccinator muscle (dashed arrow). **b** A coronal T2-weighted image at a more posterior part shows the mylohyoid muscles (arrow) and the hyoglossus muscles more medially (dashed arrow), and the masseter muscle laterally in the masticator space (dotted arrow). Submandibular glands (asterisk) are shown below the mylohyoid muscle

T2-weighted imaging is useful for anatomy: fat has a very high signal, free fluid has a high signal, soft tissues such as lymphoid tissue and mucosa have an intermediate signal, muscles have a low signal, and cortical bone has no signal (Figs. 2, 3). Fat-suppressed T2-weighted images are very sensitive to soft tissue edema, which appears bright in these images. We use the Dixon method for fat suppression because it is robust, even in large fields of view.

T1-weighted images are good for detecting fat tissue in normal anatomy, for detecting certain substances with naturally high T1 signal (such as hemorrhage or chronic secretions), and for serving as a baseline image for the assessment of contrast enhancement following intravenous administration of a gadolinium-based contrast agent. Enhancement is best appreciated on post-contrast fat-suppressed T1-weighted images. In our opinion, gadolinium-based contrast agents are mandatory to achieve optimal accuracy, because deep neck space abscesses are often inconspicuous from adjacent regions in T2-weighted (e.g., neighboring edematous tissue) and apparent diffusion coefficient (ADC) maps from diffusion-weighted imaging (DWI) (e.g., neighboring high-cellularity lymphoid tissue).

DWI is achieved using standard echo-planar imaging (EPI) with a b-value of 1000 s/mm^2^. DWI has two functions in the neck region: first, and most critical, is to depict the purulent nature of fluid collections as having restricted diffusion (high signal on trace, low ADC values), and second, to characterize physiologically high-cellular tissue, such as lymphoid tissue in the tonsils and lymph nodes.

A practical guide on how to interpret these MRI sequences to detect specific biomarkers of neck infections is provided in Table 2. Briefly, in clinical practice, we recommend starting with axial fat-suppressed T2-weighted images (look for edema and abscesses), axial fat-suppressed post-contrast T1-weighted images (look for cellulitis and non-enhancing fluid collections), and axial ADC (look for low values consistent with restricted diffusion in purulent fluid). If abscess formation is confirmed by non-enhancing collection with low ADC and surrounding enhancing soft tissues, report the location of these abscesses (anatomical neck space, maximal diameter, shape, extension to adjacent spaces, etc.) and finish the report by reporting the extent and location of edema. Sagittal and coronal images are used to increase confidence in accurately locating abscesses and edema. Finally, report potential complications.

**Table 2:** A practical guide to relevant MRI findings

Item	Useful sequences	Finding	Implications	Caveats
Soft tissue edema	T2 Dixon (water) T1 Dixon post-Gd (water)	High signal against low signal background	In the setting of suspected neck infection, implies phlegmon/cellulitis Specific edema patterns (RPE, ME, SMSE, SLSE, VSE) indicate a more severe course of illness	Any type of inflammation (also non-infectious, such as post radiation treatment) can result in soft tissue edema
Bone marrow edema	T1 SE T2 Dixon (water) T1 Dixon post-Gd (water)	Lowering of normal high fat signal on T1 High signal against low signal background in T1 post-Gd and T2 water images	Mandibular or maxillary edema usually suggests odontogenic infection Vertebral changes may suggest spinal origin of infection	Recent dental procedures and chronic osteomyelitis may cause non-specific edema Bone marrow changes are usually reactive, and not suggestive of frank osteomyelitis Spinal bone changes may be degenerative
Abscesses	T1 SE T1 Dixon post-Gd (in-phase and water) DWI	Non-enhancing collection surrounded by tissue enhancement, with restricted diffusion (low ADC)	Low ADC in a non-enhancing collection suggests drainable abscess formation Exact localization important for guiding surgical approach	Purulent fluid may not be very T2 hyperintense Suppressed fat and non-enhancing fluid may look similarly hypointense in T1 post-Gd water images (be sure to check in-phase images as well) MRI is sensitive to even small abscesses that may be surgically insignificant Poorly enhancing, necrotic lymph nodes may mimic suppurative lymphadenitis (intranodal abscess)
Complications	All	Mediastinal extension of abscesses Venous thrombosis Airway compromise	Urgent medical or surgical treatment of complications to minimize morbidity and mortality	Mediastinal edema alone does not necessarily imply descending mediastinitis or abscess, most often reactive non-suppurative edema Venous thrombosis may be difficult to diagnose on MRI Airway narrowing in MRI is non-specific and not predictive of need for airway support

## Terminology

There are no published guidelines on the nomenclature of MRI findings in the soft tissues of the neck as there are for the musculoskeletal system [9]. In the neck area, we use terms like “cellulitis” or “phlegmon” for soft tissue edema in the context of infection. We define “infection” as either a high signal of fat-suppressed T2-weighted images suggesting edema, or a high signal of fat-suppressed post-contrast T1-weighted images suggesting abnormal tissue enhancement (these findings often overlap) (Table 2). When using the final clinical diagnosis of “infection” as the reference standard, imaging has a positive predictive value (PPV) of 0.98 [2], suggesting that MRI can very accurately confirm clinical suspicion of an acute neck infection.

**Fig. 4:**
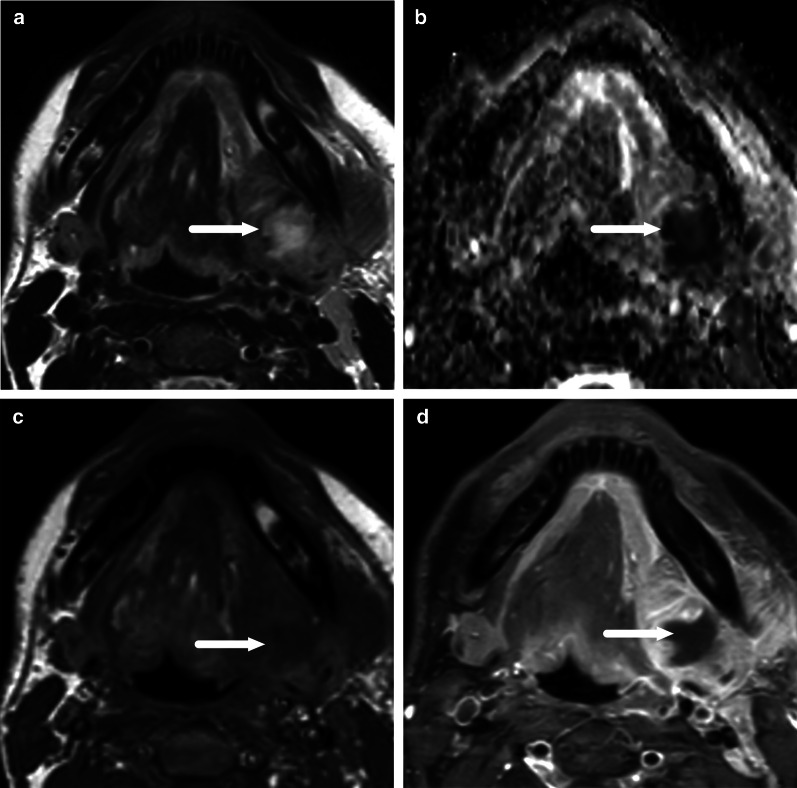
Diagnostic criteria for an abscess on MRI: axial T2-weighted (**a**), pre-contrast T1-weighted (**c**), fat-suppressed post-contrast T1-weighted (**d**) images, and an ADC map (**b**). A submandibular space odontogenic abscess (arrows) shows intermediate to high T2-signal (**a**), no enhancement after contrast injection (**c**, **d**), and restricted diffusion as indicated by low ADC values (**b**)

The MRI criteria for an abscess are an abnormal T2 iso- to hyperintense collection with low ADC, surrounded by abnormal tissue enhancement on post-contrast T1-weighted images, and no enhancement in the center (Table 2, Fig. 4). On MRI, surrounding enhancement may be ring-like or diffuse, but importantly, unlike on CT, a granulomatous enhancing capsule is not required for abscess diagnosis because the nature of the fluid content is directly assessed with DWI. This MRI definition of an abscess has a substantial interobserver agreement (Kappa 0.78) in a multi-reader setting [2]. Diagnostic accuracy in various types of abscesses is presented in the following chapters. A comparison of CT and MRI abscess criteria is given in Table 3.

**Table 3:** Diagnostic criteria for an abscess in CT and MRI

CT	MRI
Mass effect	T2 iso- or hyperintense collection
Central hypodensity	Surrounding soft tissue enhancement
Rim enhancement	No enhancement in collection
Bulging	Low ADC consistent with restricted diffusion
Surrounding edema	
Presence of air	

**Fig. 5:**
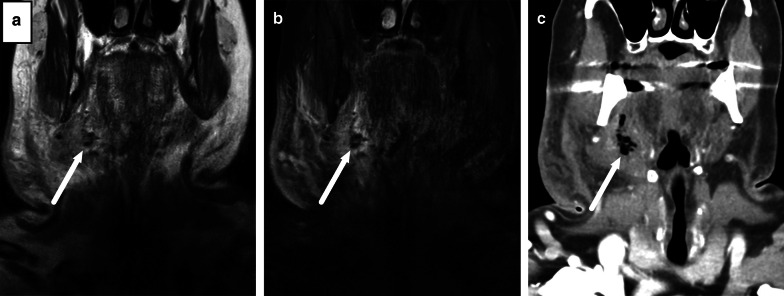
On MRI, gas appears as small foci of signal void (arrows) on coronal in-phase (**a**) and fat-suppressed T2-weighted (**b**) images but is easier to see on CT (arrow) (**c**)

Gas formation is readily detectable on CT. On MRI, gas appears as focal areas of a signal void, but is, however, often difficult to detect because images often have areas of similar low signal (Fig. 5).

## Diagnostic accuracy of diagnosing abscesses

**Fig. 6:**
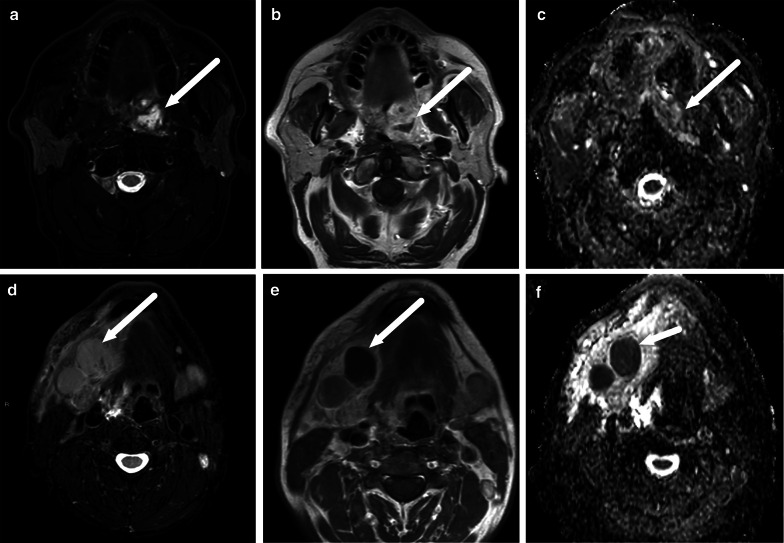
False negative (**a**–**c**) and false positive (**d**–**f**) abscesses on axial fat-suppressed T2-weighted images (**a**, **d**), post-contrast T1-weighted images (**b**, **e**), and ADC maps (**c,** **f**) (arrows). False negative, top row (**a**–**c**): in a patient with a sore throat, a T2-hyperintense non-enhancing collection with ADC values were interpreted as high rather than low (no purulence). However, surgery found an abscess. False positive, bottom row (**d**–**f**): in a patient with neck swelling, MRI shows lymphadenitis and one enlarged submandibular lymph node with no enhancement and low ADC, and suppurative lymphadenitis (intranodal abscess) was diagnosed. However, surgery demonstrated necrosis but no purulence. Image modified from ref. [2]

Because patients with an imaging diagnosis of an abscess are much more likely to undergo surgery than patients without abscesses, the diagnostic accuracy of MRI is critical. When compared with surgical findings as the reference standard (purulent fluid or abscess cavity), MRI was highly accurate in a large sample of various types of neck infections (sensitivity 0.99, specificity 0.89, positive predictive value 0.95, negative predictive value 0.98, accuracy 0.96) [2]. This diagnostic accuracy is much higher than previously reported for contrast-enhanced CT, for which the PPV is about 0.80 in clinical studies [5–7]. High diagnostic accuracy was subsequently confirmed separately in pharyngotonsillar (accuracy 0.97) [10], odontogenic (accuracy 0.92) [11], and pediatric (accuracy 0.89) [8] neck infections. False positive and negative abscess diagnoses are rare (Fig. 6) [2].

**Fig. 7:**
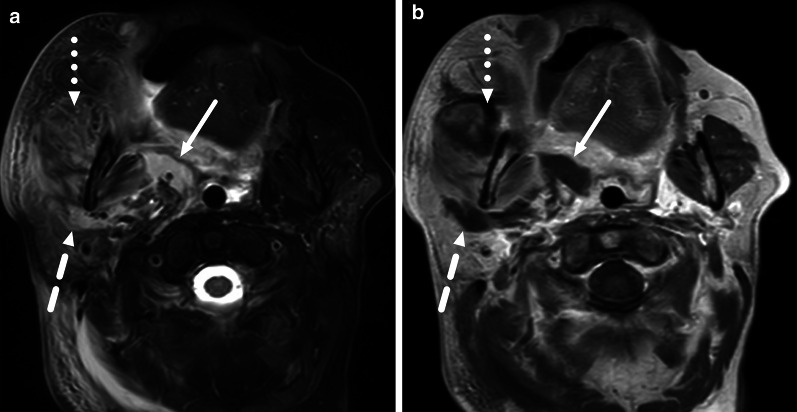
Multi-space abscess on axial fat-suppressed T2-weighted (**a**) and post-contrast T1-weighted (**b**) images. An odontogenic abscess affects the parapharyngeal (arrows), parotid (dashed arrows), and masticator (dotted arrows) spaces

**Fig. 8:**
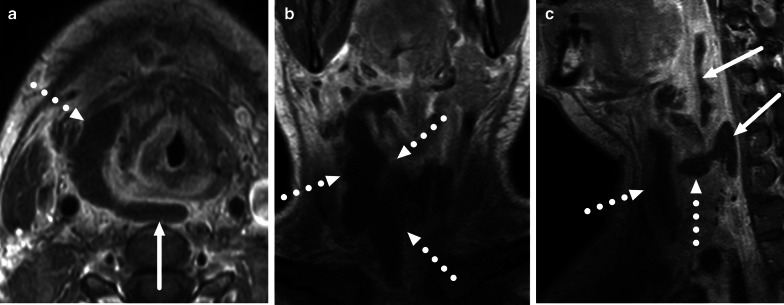
Multi-space abscess on axial (**a**), coronal (**b**), and sagittal (**c**) post-contrast T1-weighted images. A pharyngotonsillar infection was complicated by a large abscess affecting the retropharyngeal (arrows) and visceral and anterior cervical (dotted arrows) spaces

Abscesses can breach fascial planes and affect multiple neck spaces (Figs. 7, 8). Not surprisingly, larger abscesses are associated with a higher risk for ICU treatment [12], longer hospital stay [12], and higher rates of deep extension of peritonsillar abscesses [10] and extraoral surgery for odontogenic abscesses [11]. On MRI, measurement of maximal abscess diameter has high reproducibility between radiologists [12].

## Edema patterns and their clinical significance

On MRI, patients with neck infection often have reactive, non-suppurative edema, best appreciated as high signal intensity in fat-suppressed T2-weighted images and to a lesser extent post-contrast T1-weighted images. A summary of relevant edema patterns including their definitions and clinical significance is given in Table 4.

**Table 4:** Edema patterns on MRI

Anatomical compartment	Abbreviation	Definition	Clinical significance
Retropharyngeal space	RPE	Edema of at least 2 mm in anteroposterior thickness between the prevertebral muscles posteriorly and the superior pharyngeal constrictor muscle anteriorly, in at least two consecutive axial images	Predicts ICU treatment in patients with various types of neck infection, present in about half of all patients with infection More common and more caudally extending in pharyngotonsillar than in odontogenic infections
Mediastinum	ME	Edema at or below the level of the thoracic inlet, using the superior border of the manubrium sterni (anterior ME), or the first thoracic vertebra (posterior ME) as superior borders	Predicts ICU treatment and length of hospital stay in patients with various types of neck infection; present in about one-quarter of infected patients Similar prevalence in pharyngotonsillar and odontogenic infection, but the former is usually posterior and the latter usually anterior Strongest predictor of extraoral surgery in patients with odontogenic infection
Submandibular space	SMSE	Oral cavity and floor of the mouth below the mylohyoid muscle	Very common in both pharyngotonsillar and odontogenic infections; a useful indicator of acute infection but not a marker for severe illness
Sublingual space	SLSE	Oral cavity above the mylohyoid muscle	Predicts deep extension of peritonsillar abscesses
Visceral space/anterior cervical space	VSE	Infrahyoid soft-tissue space including the larynx, strap muscles, and thyroid, also including the anterior cervical space between the sternocleidomastoid muscle and the carotid space	Predicts both deep extension of peritonsillar abscesses and extraoral surgery in patients with odontogenic infections at the univariate level

**Fig. 9:**
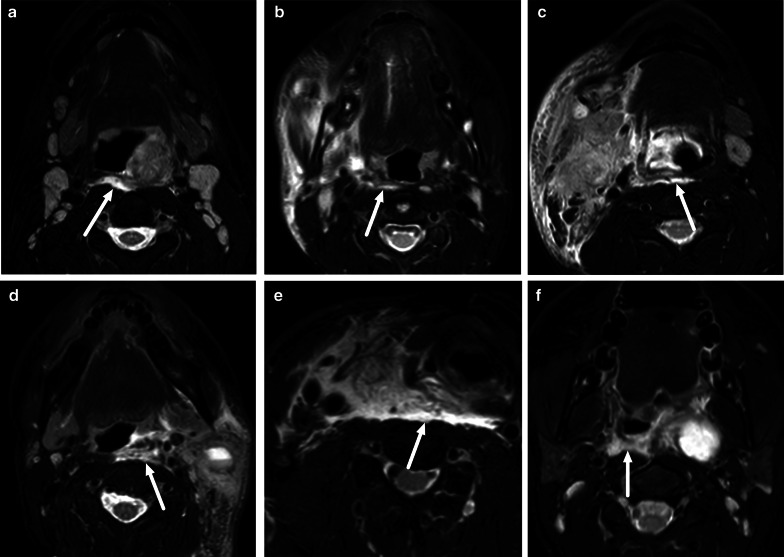
Examples of RPE (arrows) on axial fat-suppressed T2-weighted images in different patients (**a**–**f**) with infections of various origins. Image modified from ref. [12]

**Fig. 10:**
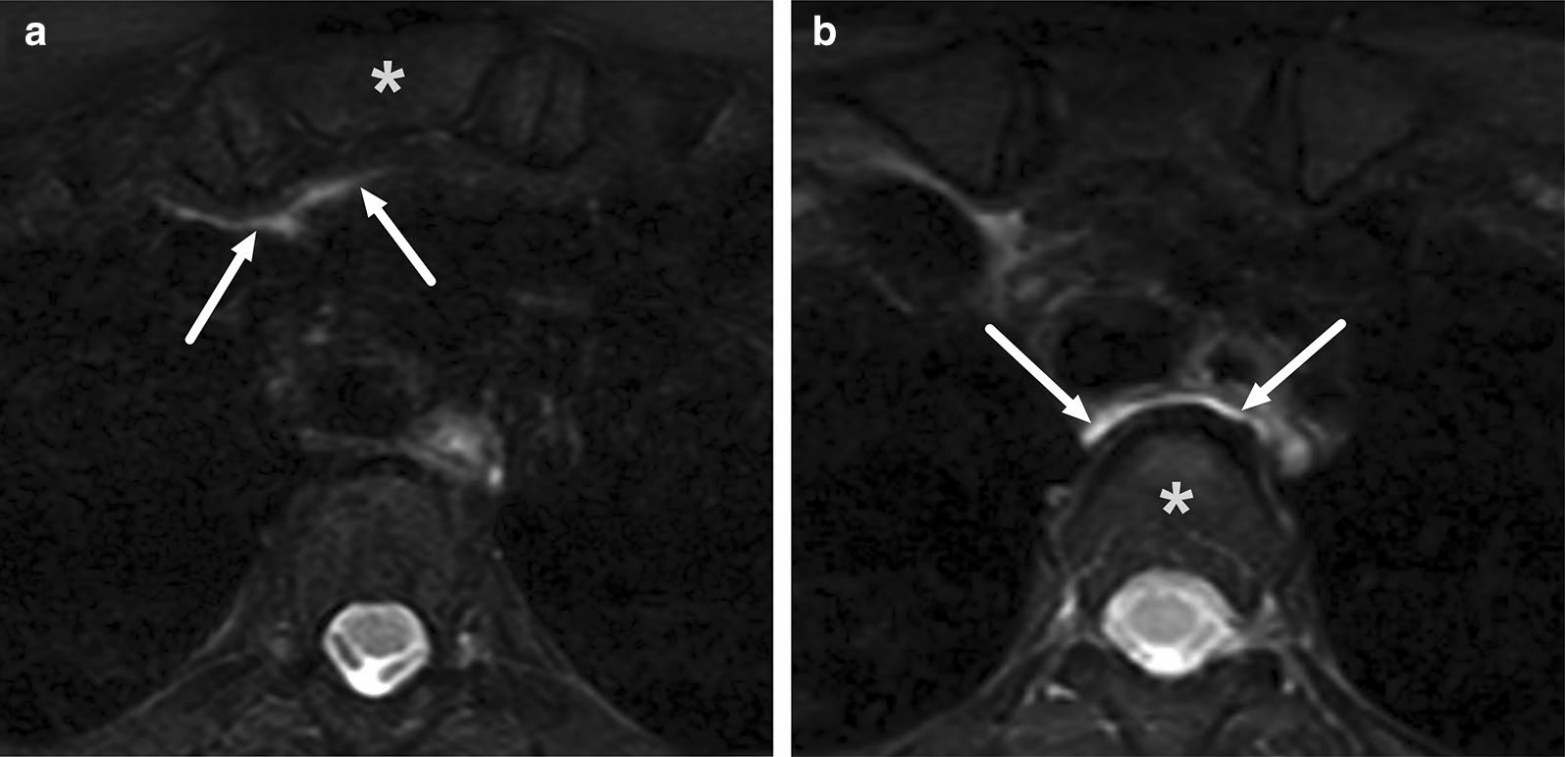
Examples of ME (arrows) on axial fat-suppressed T2-weighted images in two patients. Anterior ME (**a**) extends to the level of the sternum (asterisk), whereas posterior ME (**b**) is seen at or below the first thoracic vertebra (asterisk, in this patient the third thoracic vertebra). Image modified from ref. [12]

**Fig. 11:**
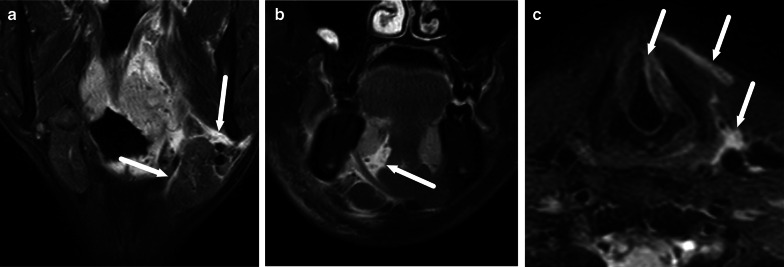
Examples of SMSE (**a**), SLSE (**b**), and VSE (**c**) on coronal (**a**, **b**) and axial (**c**) fat-suppressed T2-weighted images (arrows)

**Fig. 12:**
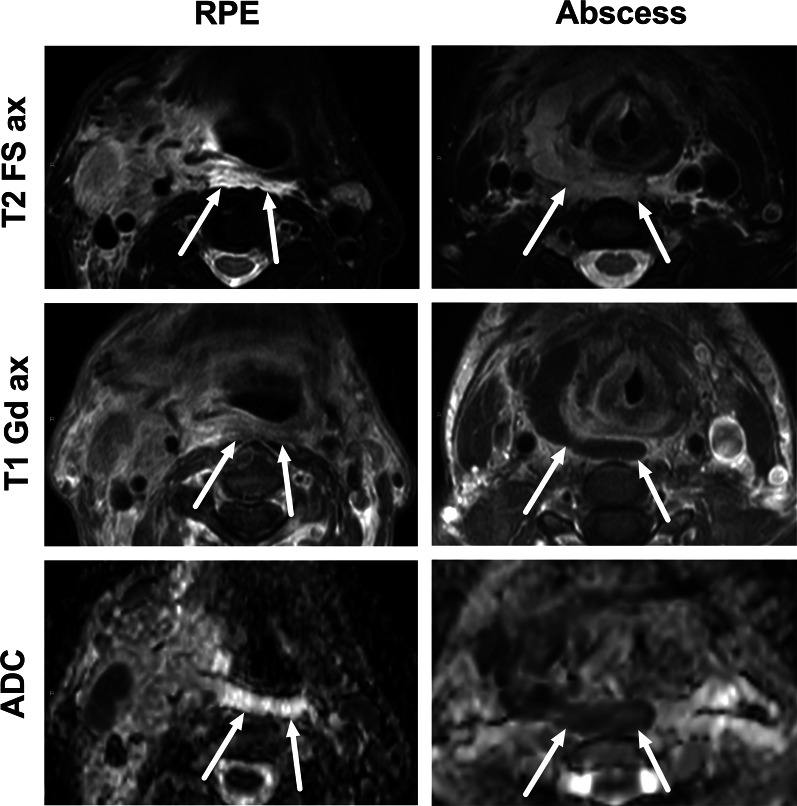
Differentiating between RPE and a retropharyngeal abscess: RPE is usually very bright on T2-weighted images, whereas abscess has an intermediate signal. Enhancement of RPE is variable, whereas an abscess does not enhance. Finally, the key is ADC: bright in RPE, dark in abscess. Arrows denote collections. Image modified from ref. [12]

One pattern of reactive edema, retropharyngeal edema (RPE), has been previously described in various neck diseases, including acute infection [13–16] (Fig. 9). In addition to RPE, soft tissue edema often reaches the level of the mediastinum in various types of neck infections, termed mediastinal edema (ME) [12] (Fig. 10). Three additional edema patterns in pharyngotonsillar and odontogenic neck infections have been standardized and validated: submandibular space edema (SMSE), sublingual space edema (SLSE), and visceral space edema (VSE) [10] (Fig. 11). These edema patterns seem to convey valuable information about the severity of the illness. They do not represent treatable fluid collections or abscesses (Fig. 12), but rather imaging biomarkers of the intensity of the disease. In a severe illness, widespread edema has crossed various anatomical boundaries between soft-tissue compartments, although we do not yet know whether this happens directly or via the lymphatic system. Detection of these edema patterns has a substantial interobserver agreement [10, 12].

RPE is present in about half of all patients with neck infections of various origins [12]. Anatomically, the retropharyngeal space is bounded anteriorly by the superior constrictor muscle and buccopharyngeal fascia, and posteriorly by the prevertebral fascia. Differentiation between true retropharyngeal space and “danger” space (alar space) has been proposed based on division by the alar fascia [17, 18], the clinical implication being that the retropharyngeal space ends at the upper thoracic level, whereas the danger space is a gateway into the lower parts of the mediastinum. In practice, these spaces cannot be reliably differentiated radiologically in the suprahyoid neck, and therefore we consider the term “retropharyngeal” to denote both these subdivisions. In patients with neck infections of various etiologies, RPE was the strongest predictor of the need for intensive care unit (ICU) treatment with a nearly ten-fold increase in risk, even when other imaging outcomes (such as abscess size) and clinical variables (such as CRP) were taken into account [12]. RPE occurred more often and extended more caudally in pharyngotonsillar than in odontogenic infections, whereas prevalence was similar between adults and children [12].

In contrast, ME is seen less often (26%) in patients with neck infection [12]. ME occurs in two forms: anterior and posterior. Anterior ME is usually a direct continuation of edema in the visceral space and anterior cervical space, whereas posterior ME is a caudal continuation of RPE. Together with RPE, ME predicts the need for treatment in the ICU, although a more significant effect was seen with the length of hospital stay [12]. A similar prevalence of ME has been found in the two most common types of infections, pharyngotonsillar and odontogenic, although ME is usually posterior in pharyngotonsillar infections (continuation of RPE) and anterior in odontogenic infections (continuation from visceral and anterior cervical spaces). ME has a comparable prevalence in adults and children. Interestingly, the effects of RPE and ME are more pronounced in odontogenic infections than in pharyngotonsillar infections, reflecting the different natures of these infections. A practical implication is that widespread edema, especially ME, should be carefully evaluated in patients with tooth infections. Finally, ME is also the strongest predictor of extraoral surgery in patients with odontogenic infections [11].

SMSE, SLSE, and VSE are more localized forms of soft tissue edema that are important for both pharyngotonsillar [10] and odontogenic [11] infections. SMSE occurs in the oral cavity and floor of the mouth below the mylohyoid muscle and is highly prevalent in both types of infections (92% and 96%, respectively). Thus, SMSE serves as a useful indicator of an acute infection in the oral cavity or oropharynx but is not an indicator of a severe illness. In contrast, SLSE, which occurs in the oral cavity above the mylohyoid muscle, is much less prevalent in pharyngotonsillar (24%) than in odontogenic (77%) infections and helps predict the deep extension of peritonsillar abscesses in the former group [10]. VSE is often a direct continuation of SMSE from above, extending to the visceral space and anterior cervical space soft tissues. It is equally common in pharyngotonsillar (72%) and odontogenic (76%) infections: at the univariate level, VSE helps predict both deep extensions of peritonsillar abscesses [10] and extraoral surgery in patients with odontogenic infections [11].

## Main types of acute neck infections

### Pharyngotonsillar infections

**Fig. 13:**
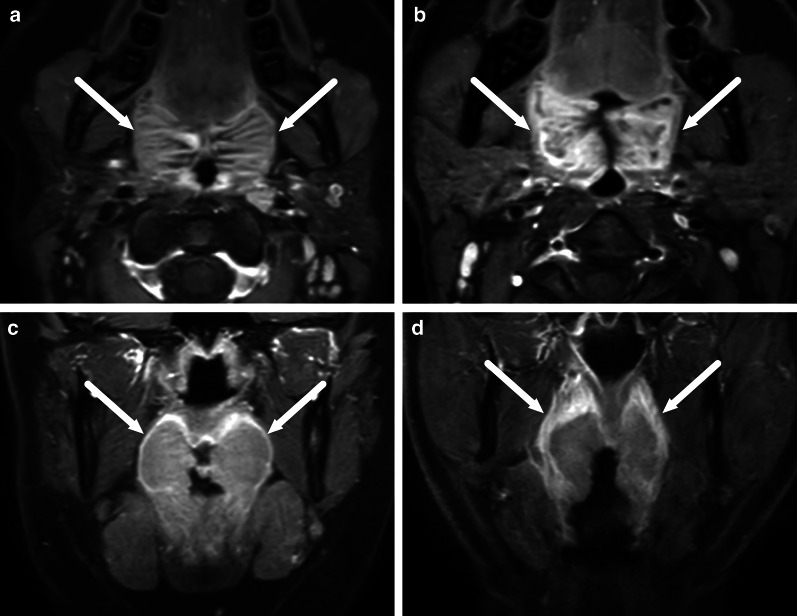
Tonsillitis/peritonsillitis on axial (**a**, **b**) and coronal (**c**, **d**) post-contrast T1-weighted images in two patients (**a** and **c**, **b** and **d**). Note the striped appearance of the tonsils (arrows in **a**), peritonsillar enhancement (arrows in **b**–**d**), and no abscess

Tonsillitis (or pharyngotonsillitis) is an acute viral or bacterial infection of the oropharynx. The imaging characteristics of tonsillitis are edema and enhancement of the palatine tonsils and surrounding oropharyngeal mucosa, but no abscess formation (Fig. 13). Surrounding enhancement is sometimes called peritonsillitis. Occasionally, the palatine tonsils have a striped appearance on post-contrast T1-weighted images, due to fluid in the tonsillar crypts (Fig. 13). These stripes should not be mistaken for small crescent-shaped abscesses.

**Fig. 14:**
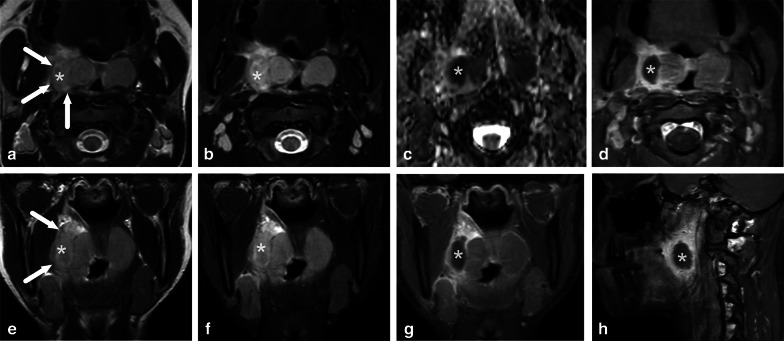
Lateral uncomplicated PTA in a patient with a sore throat. Axial and coronal T2-weighted images without (**a**, **e**) and with (**b**, **f**) fat suppression demonstrate an abnormal collection (asterisk) with intermediate T2-signal between the tonsil and a swollen pharyngeal constrictor muscle (arrows). Axial (**d**), coronal (**g**), and sagittal (**h**) fat-suppressed post-contrast T1-weighted images show a centrally non-enhancing fluid collection, and low ADC (**c**) suggests purulence. PTA was found upon tonsillectomy

**Fig. 15:**
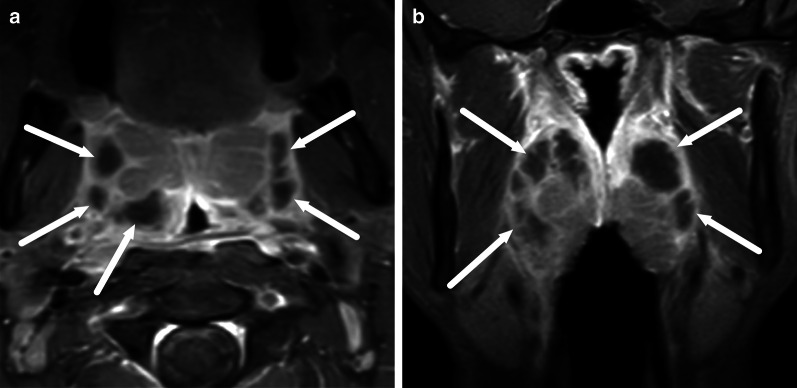
A case of multi-locular bilateral PTAs on axial (**a**) and coronal (**b**) fat-suppressed post-contrast T1-weighted images as indicated by arrows

The peritonsillar space refers to the potential space between the palatine tonsil capsule and the superior constrictor muscle, bounded anteriorly by the palatoglossus muscle (anterior tonsillar pillar) and posteriorly by the palatopharyngeus muscle (posterior tonsillar pillar) (Fig. 2). Therefore, peritonsillar space is in the pharyngeal mucosal space by definition. Peritonsillar abscess (PTA) is a collection of purulent fluid in peritonsillar space, often as a complication of tonsillitis [19] or resulting from minor salivary gland infection [20, 21] (Figs. 14, 15). PTA can be found anywhere in the peritonsillar space. Compared with other locations, superiorly confined PTAs (43% of all, defined relative to the craniocaudal midpoint of the palatine tonsil), occur in younger patients, are more common in females than in males, are smaller, have lower rates of deep extension, and patients have a shorter hospital stay [10]. Conversely, larger PTAs are associated with a more complicated course of illness. Technically, a simple PTA is not considered a deep neck infection and most cases can be managed on clinical grounds with local incision and drainage, without the need for imaging. However, in patients with PTA in whom incision yields no pus (“dry tap”), imaging can help define the extent of infection and accurate localization of abscess. For example, posterior and inferior PTA may not be drainable by simple superior incision. Imaging can also help in planning acute phase tonsillectomy.

Intratonsillar abscess (ITA) has been described as a pus collection within the tonsillar parenchyma [22] with a more favorable prognosis than PTA. By definition, an ITA would be surrounded by parenchymal tissue and would not breach the tonsillar capsule and touch the superior constrictor muscle. Previous CT studies have shown some evidence for ITAs [23–27]. However, MRI would be best suited for differentiating whether a tonsillar abscess is surrounded by tonsillar parenchyma, consistent with an ITA. In a cohort of 132 patients with acute pharyngotonsillar infections, ranging from mild tonsillitis to multi-space deep abscesses, not a single case of ITA could be identified [10]. Perhaps CT cannot differentiate between a swollen pharyngeal constrictor muscle and tonsillar parenchyma, and therefore between ITAs and PTAs, whereas MRI can accomplish this with DWI and ADC maps. It remains unclear whether ITA is a valid imaging diagnosis.

**Fig. 16:**
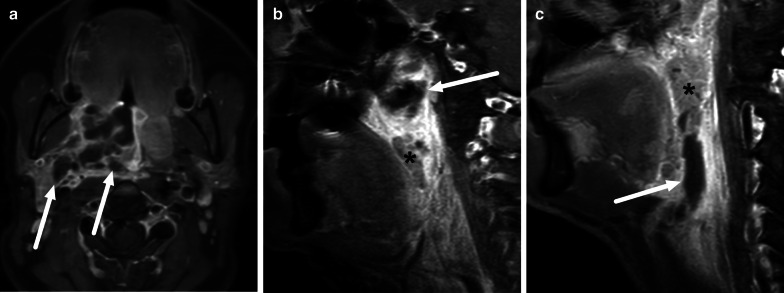
Examples of deep extension of PTAs on axial (**a**) and sagittal (**b**, **c**) fat-suppressed post-contrast T1-weighted images. Abscesses (arrows) can extend posterolaterally (**a**), superiorly (**b**), or inferiorly (**c**) from the tonsil (asterisk)

Imaging is needed when PTAs are clinically suspected of rupturing to adjacent parapharyngeal and retropharyngeal spaces. This deep extension is a serious complication, in which infection has breached the muscles and fascia that delineate the mucosal space [28]. Once in the parapharyngeal space, an infection can spread more caudally into the submandibular space, or laterally into the carotid space. Retropharyngeal abscesses may reach the mediastinum. Deeply extending disease can also lead to necrotizing fasciitis, a rapidly progressing and life-threatening soft tissue infection. The clinical implication of differentiating simple PTA and deeply extending disease is that the latter usually requires surgery in the operating room. In PTAs, purulent fluid often has a mixed intermediate signal intensity on T2-weighted images, and therefore they may be difficult to differentiate from adjacent edematous tissue. In the same manner, low ADC in purulent fluid resembles that of neighboring high-cellular lymphoid tissue in the tonsil. Therefore, post-gadolinium T1-weighted images are likely needed to accurately define pharyngotonsillar abscesses, although the magnitude of improvement in diagnostic accuracy following the usage of an intravenous contrast agent is currently unknown. Muscles and fascia between mucosal space and parapharyngeal space are not reliably discerned in all cases due to surrounding edema, therefore differentiation between PTA and deeply extending abscesses is not always easy. By definition, deep abscesses may extend more caudally, beyond the inferior aspect of the peritonsillar space. When compared to surgical findings as the reference standard, MRI has high overall accuracy (0.89) and substantial interobserver agreement (Kappa 0.75) for detecting deeply extending pharyngotonsillar abscesses [10] (Fig. 16). In addition, high CRP values, high abscess volume, and SLSE significantly predicted deep extension in a multivariate statistical model [10].

### Odontogenic (maxillofacial) infections

**Fig. 17:**
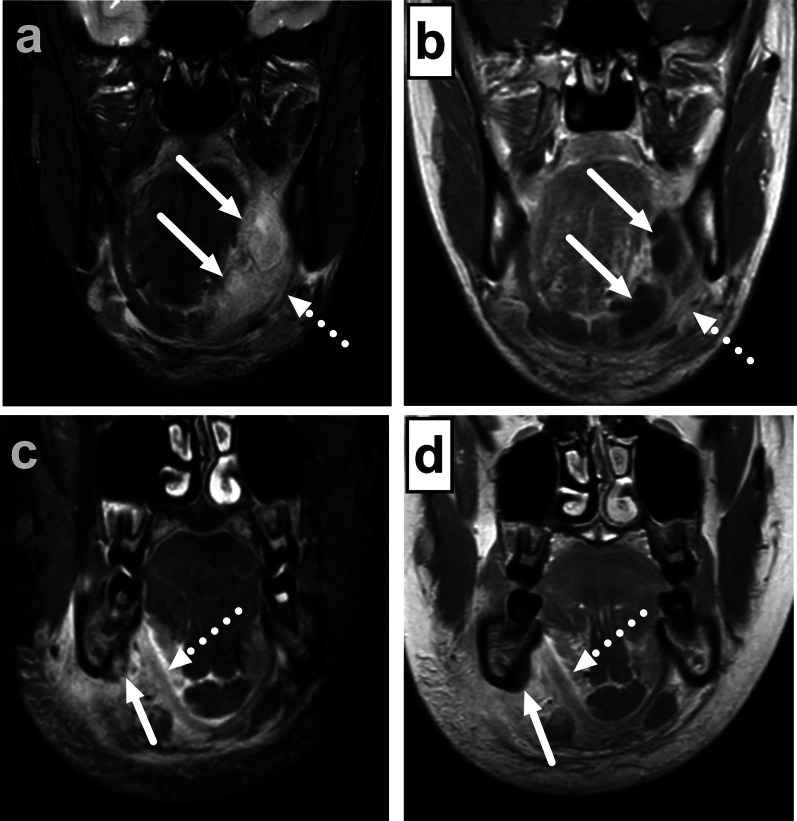
Odontogenic maxillofacial infections in two patients (**a** and **b**, **c** and **d**). Coronal fat-suppressed T2-weighted (**a**, **c**) and post-contrast T1-weighted (**b**, **d**) images show sublingual abscesses (arrows in **a** and **b**) above the mylohyoid muscle (dotted arrow), and a submandibular space subperiosteal abscess (arrow in **c** and **d**) below the mylohyoid muscle (dotted arrow). Image modified from ref. [11]

**Fig. 18:**
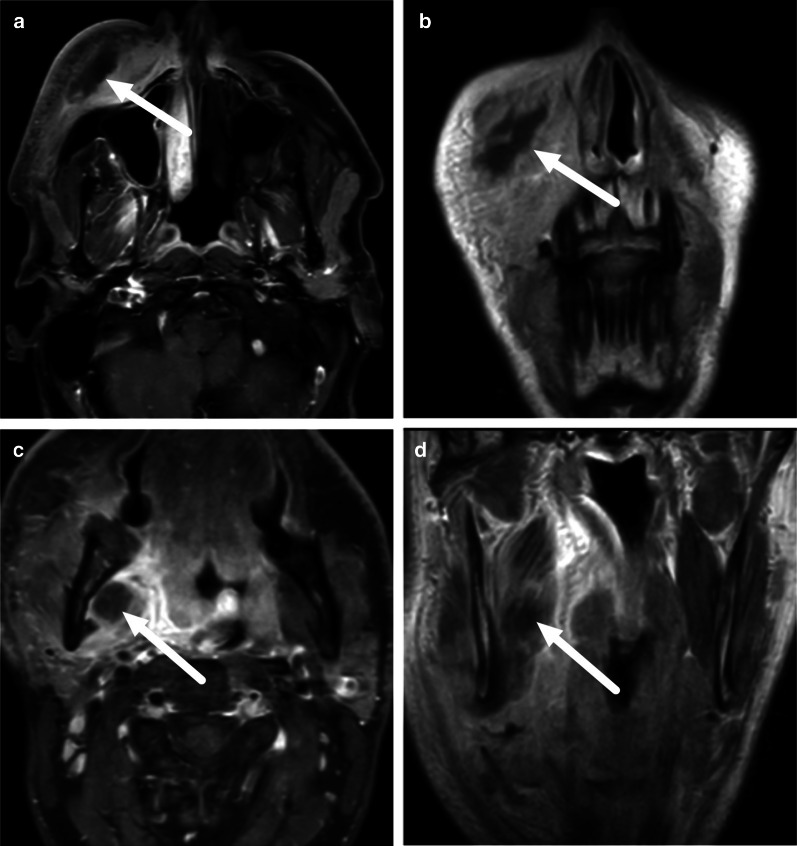
Odontogenic maxillofacial infections in two patients (**a** and **b**, **c** and **d**). Axial fat-suppressed (**a**, **c**) and coronal (**b**, **d**) post-contrast T1-weighted images show buccal space (**a**, **b**) and masticator space (**c**, **d**) abscesses (arrows). Image modified from ref. [11]

Infections in the oral cavity can be divided into sublingual and submandibular based on the superior and inferior location relative to the mylohyoid muscle, respectively (Figs. 3, 17) [29]. Often, edema comprises both compartments, and abscesses can also traverse the anatomical borders either through the muscle, via anatomical defects of the muscle, or behind the posterior free edge of the muscle, where these compartments communicate freely. These infections are most often of odontogenic origin, or, more rarely, complications of sialadenitis. Many patients have a recent history of a previous procedure, such as tooth extraction. The clinical implication of defining the exact anatomical location is that sublingual abscesses can often be drained intraorally, whereas submandibular abscesses are drained extraorally and transcervically (through the skin of the neck). Among these patients, high CRP, high WBC, larger abscesses, and the presence of RPE, ME, VSE, and SLSE predicted extraoral surgery, among which ME was the strongest predictor at the multivariate level [11]. In addition to sublingual and submandibular spaces, odontogenic abscesses can involve the buccal and masticator spaces (Fig. 18). Maxillary third molar infections can even cause parapharyngeal abscesses.

**Fig. 19:**
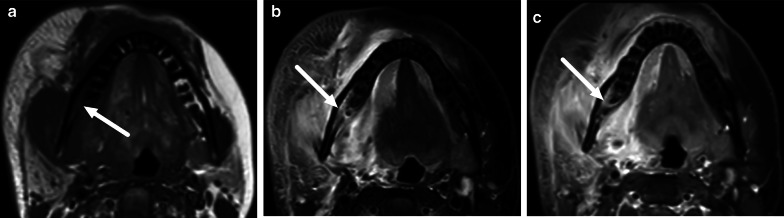
Bone marrow edema as evidenced by low T1-signal (**a**) and high signal on fat-suppressed T2-weighted (**b**) and post-contrast T1-weighted (**c**) axial images (arrows) reliably suggest an odontogenic origin of infection. Image modified from ref. [11]

An odontogenic origin of neck infections is usually suggested by signal changes in maxillofacial bone marrow (mandible or maxilla), present in about 80% of these patients (Fig. 19) [11]. Bone marrow edema is consistent with acute osteomyelitis [9], although it does not necessarily indicate bone destruction, sequestration, or bone abscess, as is often associated with this term in the mandible. Bone marrow changes have a substantial interobserver agreement.

Subperiosteal abscesses may be particularly difficult to diagnose because the non-enhancing T1-hypointense collection is immediately adjacent to cortical bone, which has no signal (Fig. 17). Careful examination of post-contrast T1-weighted images, especially in axial and coronal orientation, is critical for accurate detection. Intermediate to high T2 signal intensity may also be helpful.

**Fig. 20:**
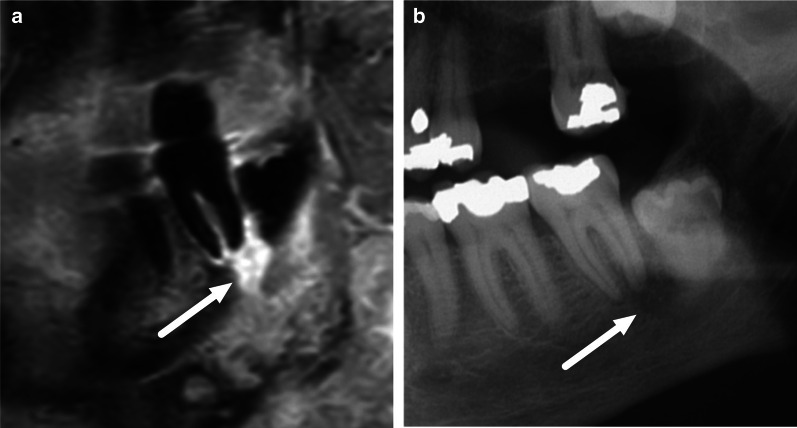
Periapical enhancement (arrow) at the tooth responsible for the neck infection, on a sagittal fat-suppressed post-contrast T1-weighted image (**a**). Radiography showed corresponding periapical radiolucency (arrow) consistent with an infection (**b**)

MRI is sometimes considered inferior to CT in demonstrating the causative tooth in odontogenic infections. Although cortical bone is devoid of MRI signal, periapical soft tissue changes, such as enhancement of infectious tissue, may help pinpoint the causative tooth (Fig. 20). These periapical changes were seen in the majority of patients with odontogenic neck infections with an unknown causative tooth. In a multi-reader setting, radiologists were able to accurately and reliably pinpoint the causative tooth within a margin of error of one tooth when the final clinical assessment was used as the reference standard [11].

### Pediatric neck infections: especially retropharyngeal and lateral lymphadenitis

Because many younger children require sedation or anesthesia for MRI, choosing an appropriate emergency imaging method for a suspected neck infection is a balance between the potential harm of radiation and the possible immediate or long-term harm related to anesthesia [30]. About a third of children required sedation with spontaneous breathing, whereas the minority required general anesthesia and intubation [8].

Overall, the diagnostic accuracy of MRI and the clinical significance of MRI biomarkers (such as edema patterns) are similar between adults and children [8]. However, there are differences between adults and children in the anatomical proportions of the neck, as well as in the proportional distribution and function of lymph nodes, resulting in somewhat differing profiles of types of neck infections that require emergency imaging. Children have a higher number of retropharyngeal infections and lateral lymphadenitis, and fewer pharyngotonsillar and odontogenic infections, compared with adults [8].

**Fig. 21:**
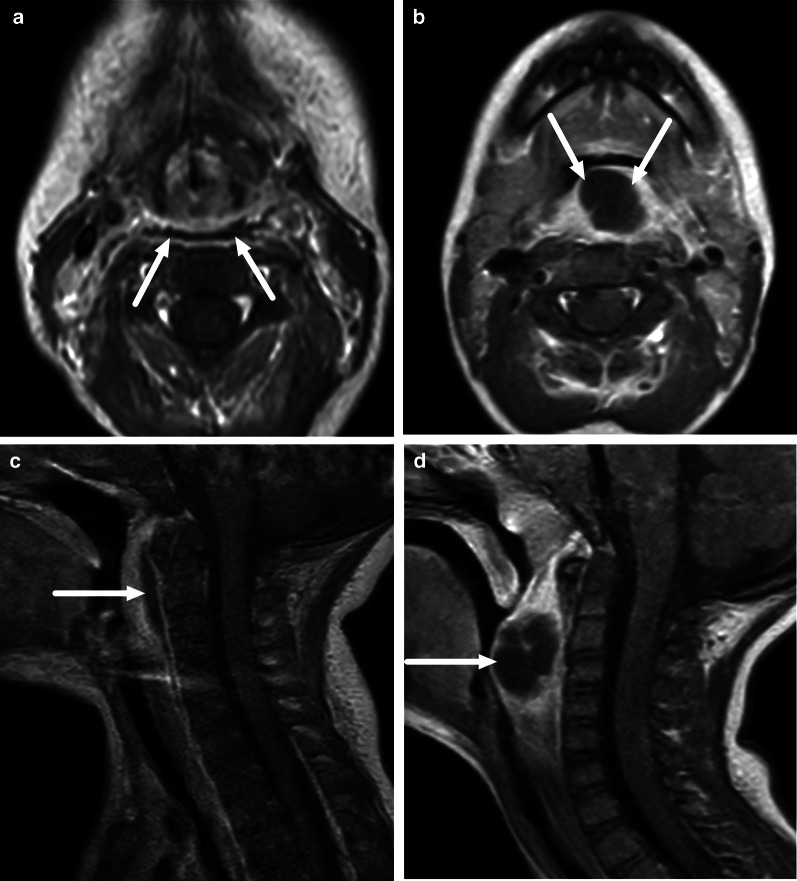
Retropharyngeal abscesses (arrows) on axial (**a**, **b**) and sagittal (**c**, **d**) post-contrast T1-weighted images in two children (**a** and **c**, **b** and **d**). The first (**a**, **c**) represent a true retropharyngeal abscess as a thin collection between fascial planes, whereas the second (**b**, **d**) is a focal collection due to suppurative lymphadenitis. Image modified from ref. [8]

Retropharyngeal infections in children can be “true” deep infections bound by fascial planes of the deep space, or suppurative lymphadenitis (nodal abscess) (Fig. 21). The key to differentiating these on imaging is that the former is often a long and thin collection in the midline, whereas the latter is a rounded collection off-midline (paramedian). In cases with true abscess or considerable infrahyoid extension of edema, imaging of the mediastinum may be warranted.

**Fig. 22:**
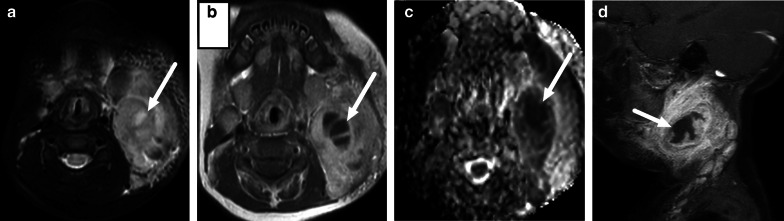
Lateral superficial suppurative lymphadenitis in an infant. Lymphadenopathy and surrounding edema are shown on an axial fat-suppressed T2-weighted image (**a**), and a non-enhancing center on post-contrast axial (**b**) and fat-suppressed sagittal (**d**) T1-weighted images with low ADC (**c**) (arrows). Imaging was consistent with suppurative lymphadenitis. Purulence was found during surgery and *Staphylococcus aureus* in pus cultures. Image modified from ref. [8]

Lateral lymphadenitis in small children can cause large masses, especially in *Staphylococcus aureus* infections (Fig. 22). These large masses are often surrounded by wide-spread soft tissue edema. Non-enhancement within the infected lymph nodes should be carefully evaluated and correlated with DWI for the accurate diagnosis of intranodal abscesses.

### Miscellaneous infections

**Fig. 23:**
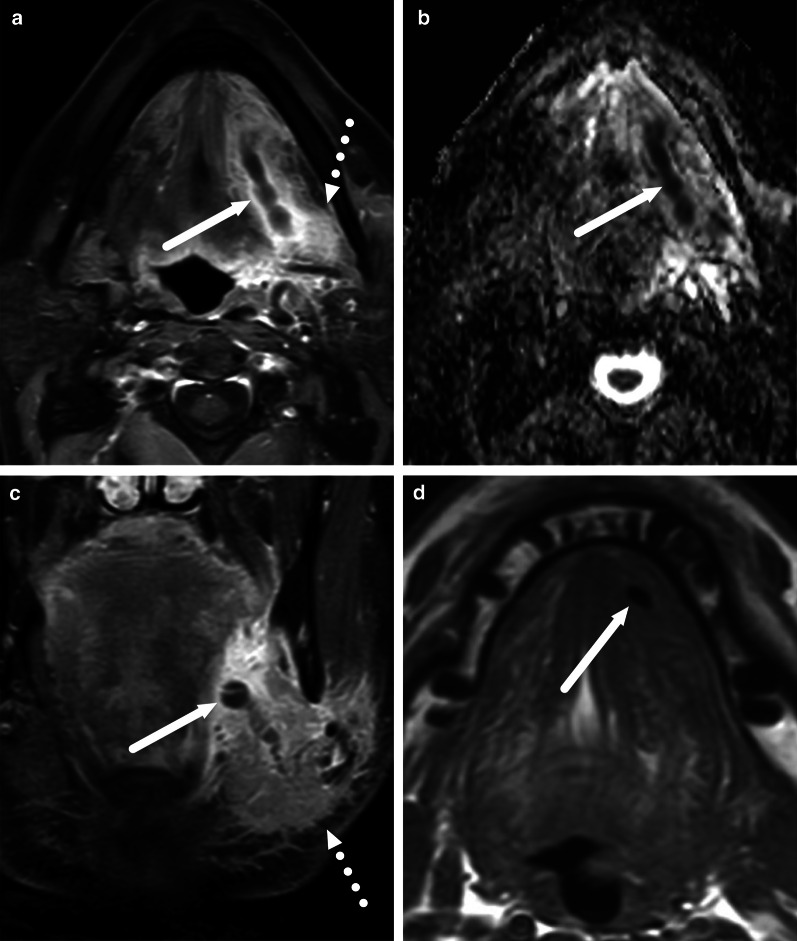
Purulent obstructive sialadenitis of the submandibular gland. Axial (**a**) and coronal (**c**) post-contrast fat-suppressed T1-weighted images show a dilated submandibular duct (Wharton’s duct) (arrow), and significant edema in and around the gland (dotted arrow). ADC map confirms that the dilated duct is filled with pus (arrow) (**b**), and axial T1-weighted imaging at a level slightly superior shows a sialolith as an ovoid area of signal void (arrow) (**d**)

Acute infectious sialadenitis can be bacterial or viral in origin [31]. Occasionally, the infection is precipitated by obstructive sialolithiasis. MRI shows edema in and around the salivary gland, and purulence can be detected using DWI in the salivary ducts (Fig. 23) or in abscesses within the glandular tissue. Sialoliths appear as focal areas of signal void (Fig. 23).

**Fig. 24:**
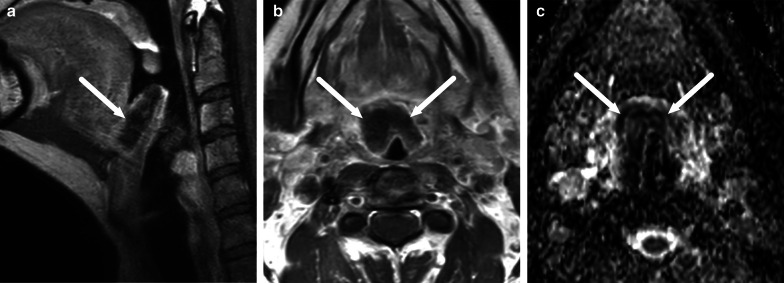
An epiglottic abscess (arrows) on sagittal (**a**) and axial (**b**) post-contrast T1-weighted images and on an axial ADC map (**c**)

Epiglottitis is nowadays rare in children due to *Hemophilus influenzae* vaccination but can still occur in adults as a serious infection [32]. MRI shows a swollen epiglottis and abscesses in and around the epiglottis (Fig. 24).

## MRI artifacts

**Fig. 25:**
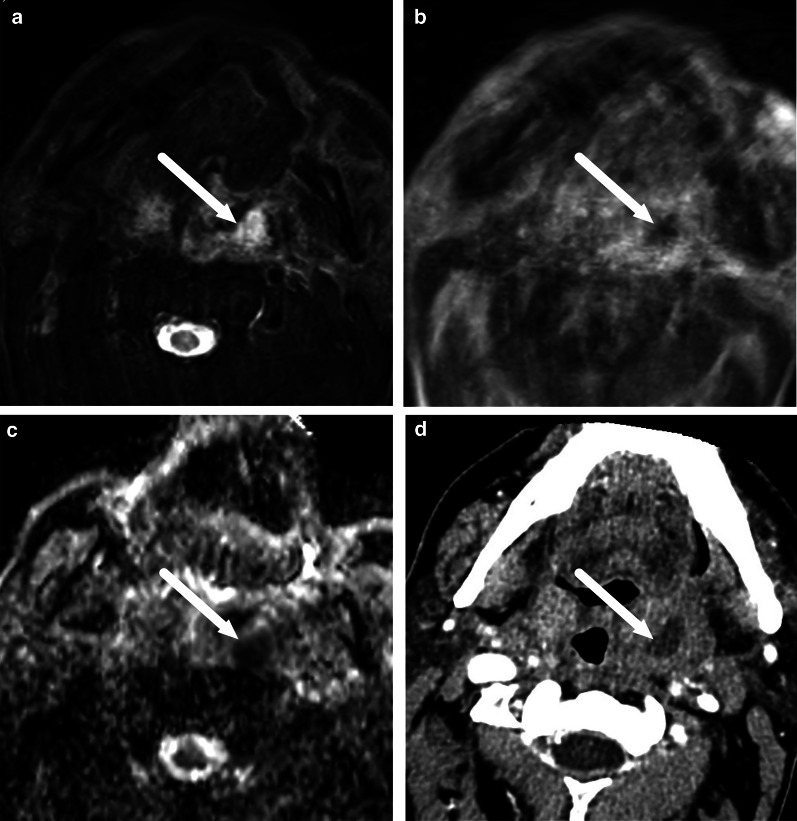
Excessive patient movement during scanning deteriorating image quality. Axial fat-suppressed T2-weighted (**a**), post-contrast T1-weighted (**b**), and ADC maps (**c**) demonstrate a left-sided PTA (arrows). The radiologist was left unsure about the true extent of the abscess in motion-degraded post-contrast images and recommended CT (**d**)

Patient movement during scanning can deteriorate image quality and lower diagnostic accuracy. In a previously published cohort, only 1% of all acute MRI scans were deemed non-diagnostic due to motion artifacts [2]. Although some preliminary diagnostics might be possible even from motion-degraded images (Fig. 25), many of these patients eventually need CT imaging.

**Fig. 26:**
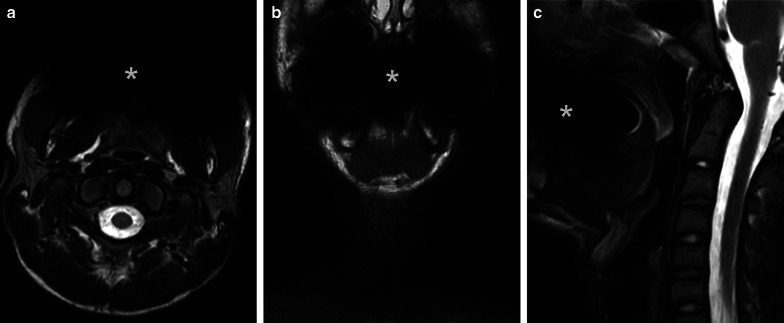
Braces causing a significant signal loss in the area of the oral cavity (asterisk) in axial (**a**), coronal (**b**), and sagittal (**c**) T2-weighted images

Ferromagnetic foreign materials can cause susceptibility artifacts and signal loss. This is particularly relevant for oral cavity imaging, where dental hardware can cause problems [33]. Luckily, in clinical practice, these artifacts are rare and usually do not cause significant problems in the diagnostic process [11]. Instead, braces can cause detrimental artifacts (Fig. 26).

## Complications

### Venous thrombosis

Lemierre’s syndrome, also known as post-anginal septicemia or necrobacillosis, typically involves internal jugular vein thrombophlebitis and septic fever following acute pharyngeal infections most commonly caused by *Fusobacterium necrophorum*. The causative focus mostly originates from pharyngitis or tonsillitis, accounting for over 85% of the cases of Lemierre’s syndrome [34]. High clinical suspicion is critical to diagnosing Lemierre’s syndrome: during the early stages of the syndrome, persistent high fever and neck pain with tenderness can be clues.

**Fig. 27:**
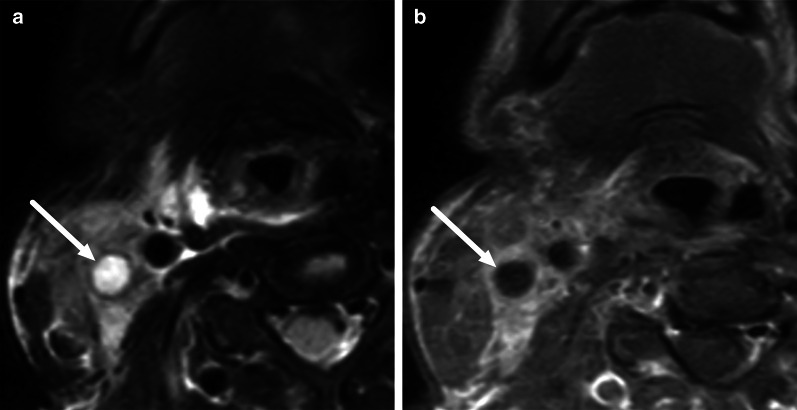
Thrombosis of the internal jugular vein (arrows) associated with Lemierre’s syndrome in a teenager with a throat infection, demonstrated on axial fat-suppressed T2-weighted (**a**) and post-contrast T1-weighted (**b**) images. Thrombosis may be difficult to detect on standard MRI sequences. Here, the lack of flow void is seen on the T2-weighted image (**a**), whereas the hypointense thrombus is difficult to separate from the flow void on the T1-weighted image (**b**)

Contrast-enhanced CT of the neck is the most commonly used diagnostic method as it can show venous thrombosis and other complications such as intracranial venous sinus thrombosis, pulmonary emboli, empyema, osteomyelitis, and brain or epidural abscess. MRI can also be used to demonstrate venous thrombosis, although it may be difficult to detect on standard sequences (Fig. 27). MRI can also better demonstrate epidural or brain abscesses.

### Airway compromise

Acute airway compromise is difficult to assess using static MRI images and must therefore be done on clinical grounds. The clinician needs to consider securing the airways before the patient is put in the scanner. Although MRI scanning of airway structures is compromised due to breathing motion artifacts and low resolution, it can be used to understand the pathophysiology of a patient's airway, especially if done to aid treatment [35]. Only a small minority of patients cannot undergo MRI because of shortness of breath [2].

### Mediastinal extension

**Fig. 28:**
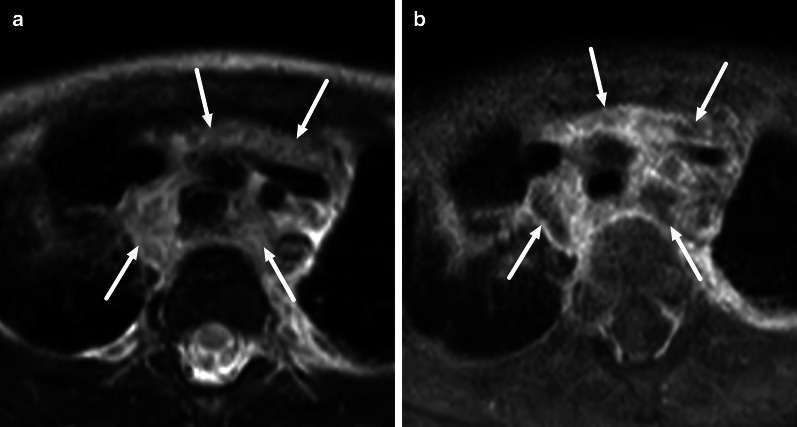
Mediastinal extension of abscesses (arrows) on axial T2-weighted (**a**) and fat-suppressed post-contrast T1-weighted (**b**) images in an infant. Note the somewhat degraded image quality at this level. The patient underwent thoracoscopy 3 days after MRI because the condition worsened during medical management

Severe infections can spread to the mediastinum, either posteriorly from the retropharyngeal space, or anteriorly through visceral and anterior cervical spaces. Subtle reactive edema (ME) should not be mistaken for treatable mediastinal disease. Accurate delineation of mediastinal abscesses is important (Fig. 28). Although small mediastinal abscesses may be treated conservatively, especially wide-spread abscesses may require surgery after a clinical multidisciplinary evaluation. Extending the imaging field of view from the neck to the mediastinum is straightforward with contrast-enhanced CT but requires more time and effort with MRI because sequences may need to be specifically tailored for the mediastinum. This requires more scanning time and radiologist supervision. In the chest area, susceptibility effects caused by interfaces between soft tissues and air cause artifacts that may degrade diagnostic performance.

### Necrotizing fasciitis

Necrotizing fasciitis of the head and neck is a rapidly progressing and life-threatening infection caused by bacteria. The infected tissue becomes necrotic as a process involving vasculitis with microthrombosis and edema of the subcutaneous tissues progresses, leading to severe systemic toxicity in the later stages. Necrotizing fasciitis of the head and neck is a rare condition, given that the incidence of 2 per 1,000,000 inhabitants per year has been reported in Denmark [36]. Patients with necrotizing fasciitis usually present with pain, swelling, crepitation, and erythema; typically, the pain is out of proportion to the swelling or erythema. Comorbidities include diabetes and liver cirrhosis. Early diagnosis and surgical intervention are key determinants of prognosis. In an uncertain case, early surgical exploration may be the best approach [37].

**Fig. 29:**
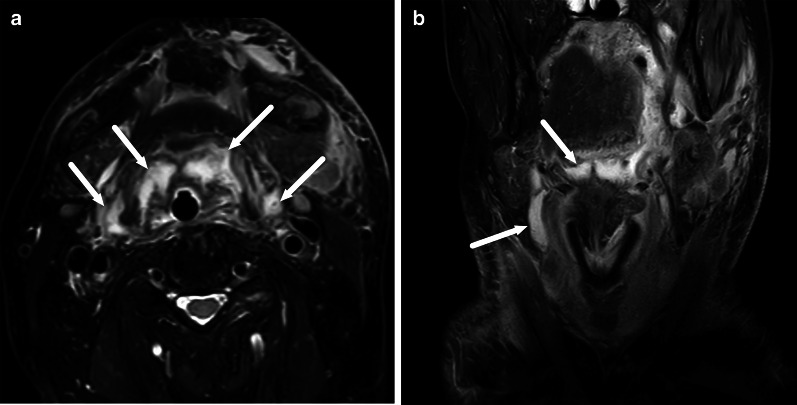
A case of fatal necrotizing fasciitis following odontogenic infection. Axial (**a**) and coronal (**b**) fat-suppressed T2-weighted images show extensive soft tissue edema, fluid along fascial planes, and multi-space abscesses (arrows)

Generally, findings of necrotizing versus non-necrotizing fasciitis may overlap. On MRI, necrotizing fasciitis in the neck is suggested by widespread edema, fluid collections along the fascial planes, and gas formation (Fig. 29).

## Imaging after surgery

**Fig. 30:**
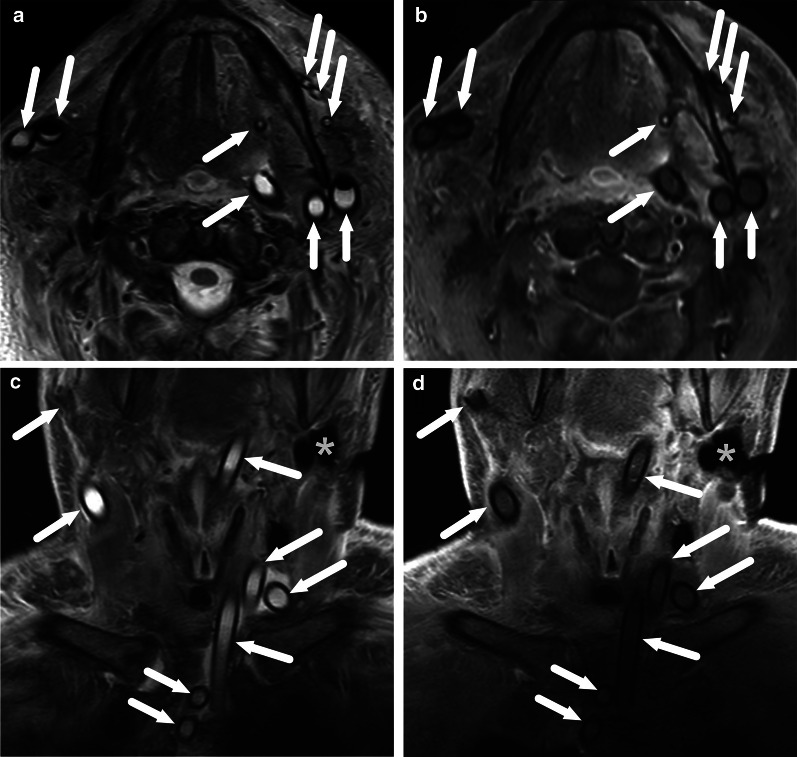
Post-treatment images of a patient with a severe neck infection, who had a total of 13 drainage tubes (arrows) and wounds intentionally left open (asterisk). Axial (**a**) and coronal (**c**) T2-weighted images and axial (**b**) and coronal (**d**) post-contrast T1-weighted images

The purposes of imaging after surgical treatment are to ensure proper placement of drainage tubes and to assess residual abscess collections. Drainage tubes are usually well visible as low-signal continuous, curvilinear structures (Fig. 30). Residual abscess collections are detected with fat-suppressed post-contrast T1-weighted images and DWI. In these cases, preoperative images are helpful.

## Mimics and difficult diagnoses

### Cystic masses

**Fig. 31:**
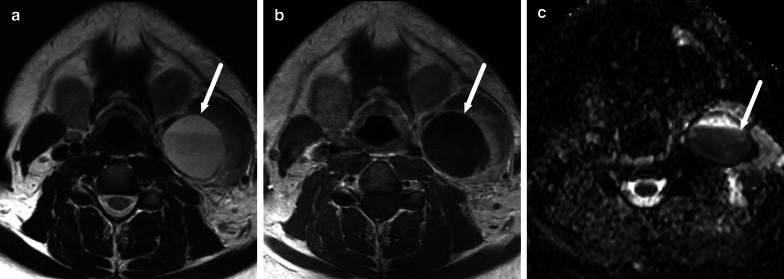
Infected second branchial cleft cyst (pathology proven). MRI shows a large cystic mass (arrows) under a swollen sternocleidomastoid muscle on axial T2-weighted (**a**) and post-contrast T1-weighted (**b**) images. ADC map shows a layering of purulent fluid (**c**), consistent with a secondary infection of a cystic mass

**Fig. 32:**
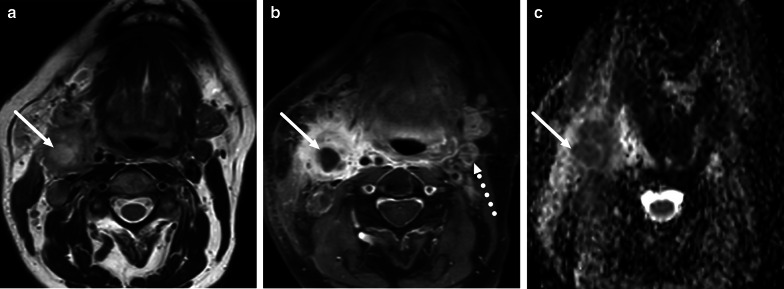
Infected necrotic lymph node metastasis (arrows) from oropharyngeal cancer misdiagnosed as suppurative lymphadenitis on axial T2-weighted (**a**) and fat-suppressed post-contrast T1-weighed images (**b**) and ADC (**c**). The patient had signs and symptoms of an acute neck infection. Low ADC suggests that the non-enhancing fluid collection is purulent. The cancer was not known at the time of imaging. Note the necrotic lymph node without signs of infection on the contralateral side (dotted arrow in **b**), suggestive of malignancy

Both benign and malignant cystic neck masses can become infected and mimic abscesses. Benign cystic masses include vascular malformations and developmental branchial cleft cysts, and, if previously undiagnosed, are often diagnosed after the acute infection has dissipated (Fig. 31). Clinically the most important malignant cystic neck mass that can be secondarily infected is the necrotic lymph node metastasis from neck mucosal space squamous cell carcinoma (Fig. 32). The importance of this caveat is increasing because of the rising incidence of cystic necrotic metastases from human papillomavirus (HPV) positive oropharyngeal carcinoma, especially in young adults. Although not secondary to underlying cystic mass, suppurative lymphadenitis refers to a collection of purulent fluid within an infected lymph node; imaging findings are similar to those of true abscess or infected cystic mass, but diagnosis can often be made based on location and general lymphadenopathy.

### Hematoma

**Fig. 33:**
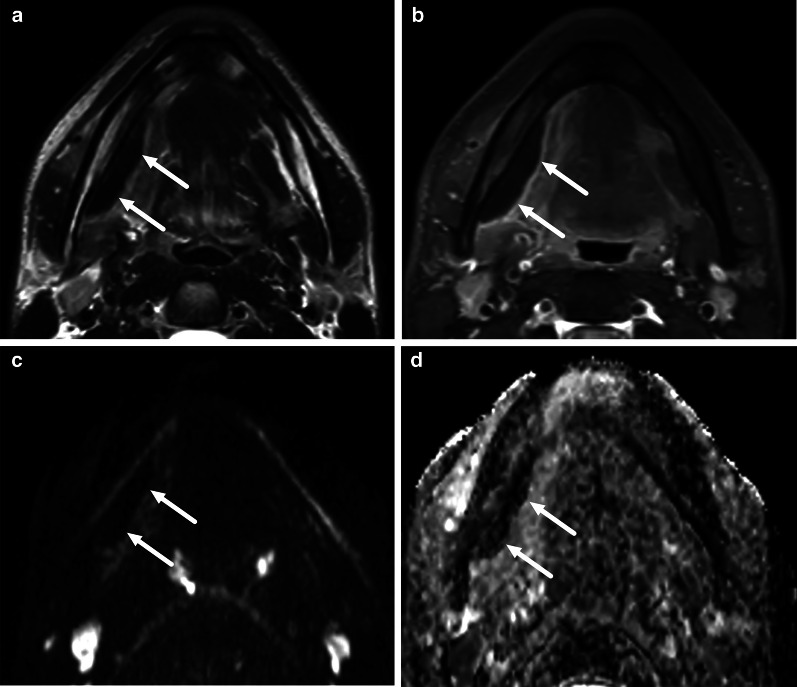
Subperiosteal hematoma after oral surgery (arrows). The collection is very hypointense on the axial T2-weighted image (**a**) which would be unusual for an abscess but mimics an abscess on the post-contrast fat-suppressed T1-weighted image (**b**). DWI of a hematoma can be misleading: the DWI trace image (**c**) shows T2 dark-through effects, whereas the ADC of the collection (**d**) looks artifactual and not like a true abscess. Surgery found hematoma

In this clinical scenario, hematomas can be spontaneous, post-traumatic, or, most commonly, post-operative (e.g., after tooth extraction). Hematomas can be mistaken for abscesses. In contrast to hematomas, however, acute neck hematomas have a low T2 signal due to susceptibility effects of blood products, whereas the T1 signal is low as it is in abscesses (Fig. 33). Trace DWI images can show dark-through T2 effects, and susceptibility effects often distort ADC modeling resulting in artifactual ADC maps (Fig. 33).

### Necrotic versus purulent lymphadenitis

**Fig. 34:**
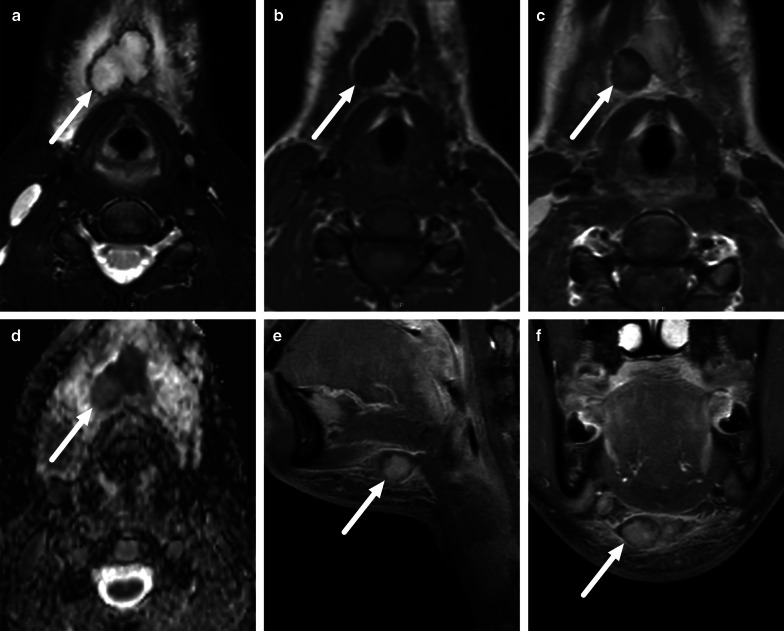
Necrotic lymphadenitis (arrows) misdiagnosed as suppurative lymphadenitis in a teenager. Lymphadenitis is seen on the axial fat-suppressed T2-weighted image (**a**) and poor enhancement on the post-contrast T1-weighted image (**c**) compared with the pre-contrast image (**b**). ADC shows restricted diffusion (**d**). The finding was misinterpreted as suppurative lymphadenitis; however, at later time points, delayed enhancement is seen (**e**, **f**), ruling out suppuration. Surgery found necrosis, but no pus. Image modified from ref. [8]

By definition, the central contents of purulent lymphadenitis (intranodal abscess) do not enhance and have restricted diffusion (low ADC). Early in the disease process, however, the lymph node can be necrotic, but not yet purulent. Necrotic lymph nodes may enhance slower than normal lymph nodes, and enhancement may not be detected if late scans (about 10 min after contrast administration) are not carefully evaluated. Thus, in early scans, necrosis and slow enhancement may be mistaken for non-enhancement. Because lymph nodes have physiologically restricted diffusion (low ADC) due to high cellularity, early necrosis may not be correctly identified and purulence may be suggested instead, possibly leading to unnecessary surgery (Fig. 34).

### Non-purulent fluid collections

**Fig. 35:**
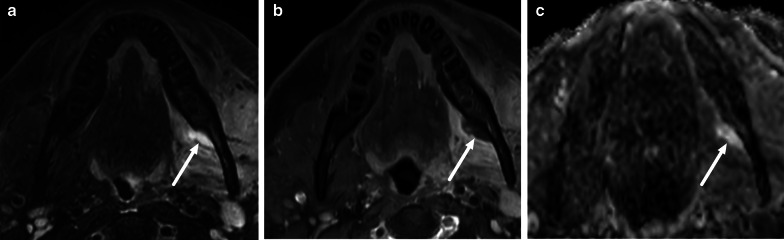
Sterile subperiosteal collection (arrows) after oral surgery on axial fat-suppressed axial T2-weighted (**a**) and post-contrast T1-weighted (**b**) images and ADC (**c**). High ADC values suggest sterile fluid rather than pus

Fluid collections that are not purulent are usually sequelae of previous operations, such as incision and drainage. The clinical question is often whether the abscess has been completely drained, or whether residual purulent collections are seen. In these patients, non-purulent fluid can have a high T2 signal, low T1 signal, and no enhancement, mimicking residual abscess at first glance. Importantly, however, these sterile collections have high ADC values on DWI, in contrast to low ADC values in purulent fluid (Fig. 35).

### Non-infectious inflammatory disease

**Fig. 36:**
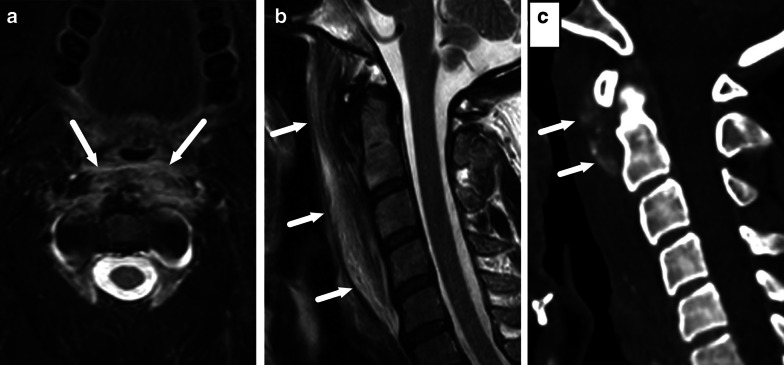
Calcific tendinitis of the longus colli mimicking a retropharyngeal infection. Axial fat-suppressed T2-weighted (**a**) and sagittal T2-weighted (**b**) images show massive swelling of the longus colli muscles and retropharyngeal tissues (arrows). A sagittal non-contrast CT (**c**) shows a pathognomonic finding of amorphous calcifications of the longus colli muscle (arrows)

Calcific tendinitis of the longus colli muscle is an inflammatory reaction to calcification in the tendon of the longus colli muscle [38]. Signs and symptoms include neck pain and stiffness, fever, and dysphagia, which can simulate those of a deep neck infection. On MRI, the longus colli prevertebral muscles and surrounding soft tissues are edematous, and patients have thick RPE mostly in the cranial part of the retropharyngeal space (Fig. 36). Amorphous calcifications within the longus colli muscle tendons may be more easily detected on CT (Fig. 36). Detecting the hypointense calcification, and, thereby, differentiating calcific tendinitis from deep neck infection and abscess formation is critical [38] because the former can be treated with anti-inflammatory drugs without any antibiotics or surgery.

**Fig. 37:**
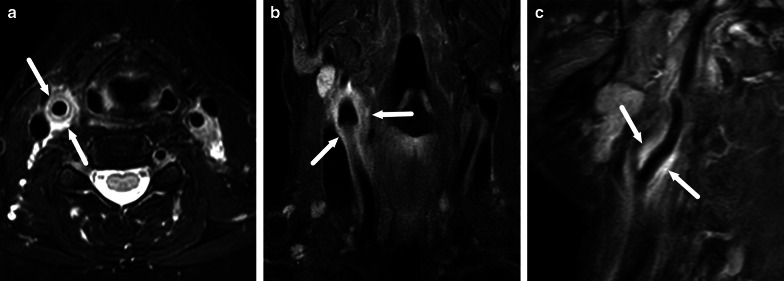
Carotidynia (TIPIC syndrome). Axial fat-suppressed T2-weighted (**a**) and coronal (**b**) and sagittal (**c**) fat-suppressed post-contrast T1-weighted images show inflammation in and around the right carotid sheath

Some patients with cervical tenderness and pain present with unclassified vascular and perivascular changes on imaging at the level of the carotid bifurcation, previously reported as carotidynia. More recently, this specific entity of idiopathic carotid inflammation has been introduced as TIPIC syndrome (transient perivascular inflammation of the carotid artery) [39]. Ultrasound appears to be a suitable examination for detecting signs of perivascular inflammation while being affordable and easily accessible. MRI shows edema in and around the carotid sheath (Fig. 37). Clinically, symptoms of TIPIC syndrome can be treated with anti-inflammatory drugs.

### Angioedema

**Fig. 38:**
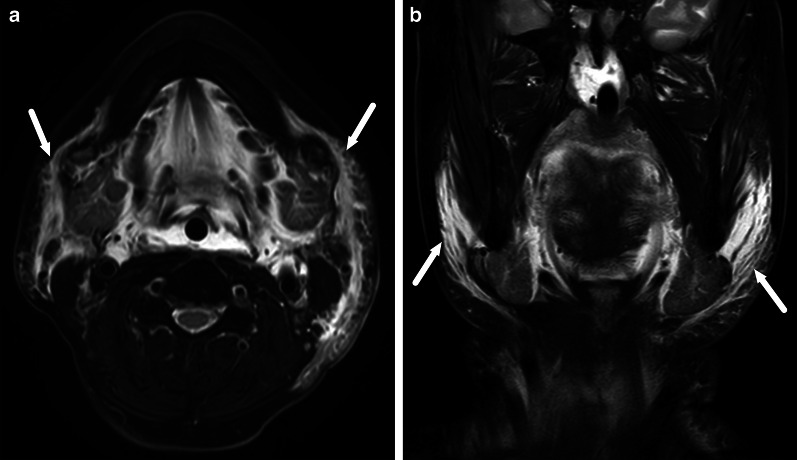
Patient with acute angioedema shows widespread edema in the neck soft tissues on axial (**a**) and coronal (**b**) fat-suppressed T2-weighted images (arrows), but no abscesses

Angioedema is a sudden swelling of the soft tissues of the face and neck that can be allergic, hereditary, or drug-induced [40]. Angioedema presents with rapid swelling and shortness of breath, and patients may quickly need airway support. MRI shows widespread soft tissue edema, especially on fat-suppressed T2-weighted images, but no signs of purulence (Fig. 38).

## A clinician’s perspective

From the point of view of the head and neck surgeon, MRI imaging in suspected acute deep neck space infections has improved diagnostic accuracy. Using CT, the hypodense area with enhancing rim can often be easily detected, but the surrounding tissue appears blurry, and delineating between an abscess and edema can sometimes be difficult. Truthfully, it was not easy to get used to this new imaging modality, and especially in the beginning, the key images provided by the radiologists were priceless. Now, after nearly 10 years of experience with good availability of acute phase MRI imaging, the clinician feels quite confident with these images even without any theoretical training in MRI imaging itself. At the end of the day, good knowledge of anatomy is the key to successful diagnosis and the visualization of the anatomy is excellent using MRI. This also serves as a reliable map for surgery. As deep neck infections can be a continuum of different phases from infectious edema to a purulent abscess, repeated imaging is sometimes crucial to evaluate the healing process or to decide when to operate or re-operate. MRI provides this information without ionizing radiation which is especially important for pediatric patients.

## Future directions

Emergency MRI is currently limited in terms of availability but might become more feasible and effective in the future due to technological advances. Possible developments for the near future include improved hardware, such as low field [41] and portable [42] MRI, as well as faster scanning using acceleration techniques [43], artificial intelligence-based reconstruction [44], and synthetic MRI sequences [45]. Thus, wider adoption of emergency MRI for acute neck infections can reasonably be foreseen.

Yet, potential bottlenecks in adopting new imaging protocols are radiologists' cognitive biases that affect their decision-making about future techniques. For example, radiologists can have a fairly robust commitment bias for CT in deep neck infections that can be difficult to overcome on an individual and systemic level [46]. Our bounded rationality and status quo bias can make us satisfied with good enough options without going through the trouble of investigating other possibly better ones [47, 48]. Any new imaging method or protocol has to overcome these several, sometimes irrational biases to become common in the field.

Improved CT methodology may close the gap in diagnostic accuracy between CT and MRI in the future. Spectral CT, including dual-energy CT, enables a more accurate assessment of tissue attenuation by detecting attenuation data of multiple different energy spectra of x-rays [49]. In head and neck diseases, dual-energy CT improves soft tissue sensitivity compared with traditional single-energy CT [50]. There is preliminary evidence for improved delineation of neck abscesses using dual-energy CT [51, 52], but a direct comparison with MRI has not been done.

## Conclusions

MRI is a nascent alternative imaging modality for acute neck infections in the emergency department. Recently, the successful implementation of MRI in the emergency department has been described, and good feasibility, high diagnostic accuracy, and derivation of clinically meaningful information from neck MRI images have been documented. These advantages pave the way for more widespread reliance on emergency neck MRI in clinical decision-making, which will ultimately benefit the patients.

## Data Availability

Data availability is not applicable to this article as no new data were created or analyzed in this study.

## Abbreviations

ADC: Apparent diffusion coefficient
CT: Computed tomography
DWI: Diffusion-weighted imaging
ITA: Intratonsillar abscess
ME: Mediastinal edema
MRI: Magnetic resonance imaging
PTA: Peritonsillar abscess
RPE: Retropharyngeal edema
SLSE: Sublingual space edema
SMSE: Submandibular space edema
VSE: Visceral space edema
